# ExtraUterine Growth Restriction (EUGR) in Preterm Infants: Growth Patterns, Nutrition, and Epigenetic Markers. A Pilot Study

**DOI:** 10.3389/fped.2018.00408

**Published:** 2018-12-20

**Authors:** Maria Giulia Tozzi, Francesca Moscuzza, Angela Michelucci, Francesca Lorenzoni, Cinzia Cosini, Massimiliano Ciantelli, Paolo Ghirri

**Affiliations:** ^1^Division of Neonatology and NICU, Department of Clinical and Experimental Medicine, Pisa, Italy; ^2^Molecular Genetics Lab, University Hospital of Pisa, Pisa, Italy

**Keywords:** extrauterine growth restriction, growth patterns, nutrition, IC1 methylation, epigenetics

## Abstract

**Background/Aims:** IntraUterine (IUGR) and ExtraUterine Growth Restriction (EUGR) may induce reprogramming mechanisms, finalized to survive before and after birth. Nutritional factors and other environmental signals could regulate gene expression through epigenetic modification, but the molecular mechanisms involved are not yet well understood. Epigenetic mechanisms could be considered as a bridge between environmental stimuli and long lasting phenotype, acquired during the intrauterine life and the first weeks of life. Our aim was to investigate the relationship between growth patterns, nutritional determinants, and epigenetic pathways.

**Methods:** We enrolled 38 newborns admitted to Neonatal Intensive Care Unit (NICU) at University Hospital of Pisa. Gestational age at birth was <34 weeks and post-menstrual age (PMA) was 36–42 weeks at discharge. We excluded infants with malformations or clinical syndromes. EUGR was defined as the reduction in weight z score between birth and discharge >1 SD. We also evaluated DNA methylation of Imprinting Centre 1 (IC1) at birth and at discharge.

**Results:** We observed a decrease in SD of weight and head circumference mainly during the first weeks of life. We found a correlation between EUGR for weight and for head circumference and an increased IC1 methylation (*p* = 0.018 and *p* = 0.0028, respectively). We observed a relationship between reduced protein and lipid intake and IC1 hypermethylation (*p* = 0.009 and *p* = 0.043, respectively).

**Conclusion:** IC1 hypermethylation could be a reprogramming mechanism to promote a catch-up growth, by means of an increased Insulin-like growth factor 2 (IGF2) expression, that may have potential effects on metabolic homeostasis later in life.

## Introduction

Extrauterine growth restriction is still a serious problem in very low birth weight preterm infants (VLBW), since the gradual improvement in neonatal intensive care has allowed the survival of newborns with an increasingly low weight and gestational age and major necessity of optimal nutrition (1, 2). In VLBW infants has been proposed an “aggressive” nutrition, to achieve a growth rate as close as possible to that of a fetus of the same gestational age (3). Adequate nutrition during the first days of life is essential to ensure to the infants a proper growth, in terms of the achievement of appropriate auxological parameters, corrected for the gestational age, and neurodevelopment (4, 5). Unfortunately, despite numerous efforts of improving the neonatal nutrition of VLBW infants, many of these do not receive an adequate nutritional supply for their increased demands, resulting in a growth restriction (2).

In many studies EUGR was defined as growth parameters (weight, length, cranial circumference) ≤ 10th percentile (z-score < -1.28) for post-menstrual age (PMA) at discharge (6). However it is more appropriate to consider EUGR as the reduction in weight z-score between birth and discharge >1 SD (7, 8). The EUGR may be present both in small for gestational age (SGA) infants at birth and in appropriate for gestational age (AGA) infants (9). However, the EUGR of a VLBW preterm newborn occurs in the same “window” in which the IUGR of the newborn at term is realized (10). Very preterm infants are exposed to the extrauterine environment in a period normally characterized by a rapid intrauterine growth. A real shift in energy expenditure is necessary to survive in this premature and unexpected postnatal life (11).

Nutritional factors and other environmental signals could regulate gene expression through epigenetic modification (12–14), but the molecular mechanisms involved are not yet well understood. Epigenetic mechanisms could be considered as a bridge between environmental stimuli and long lasting phenotype acquired during the intrauterine life and the first weeks of life (15). These mechanisms are plastic and respond to environmental signals, including diet (15). DNA methylation is a key component of epigenetic network and nutrients may modify the pattern of DNA methylation (16, 17). One of the most important mechanisms with an epigenetic control is genomic imprinting. A key region, involved in the control of growth and development, is located in chromosome 11 at p15. In this site there is IC1, an independent imprinting control region or imprinting centre (IC), that regulates IGF2 gene expression, an important growth stimulator gene, and H19, a gene with unknown function (18). Alterations of the imprinting mechanism at this point could cause to phenotypes of altered growth (19, 20) as observed in Beckwith-Wiedemann Syndrome, in which IC1 hypermethylation determines overgrowth (21), and in Silver Russell Syndrome, in which IC1 hypomethylation causes growth restriction (22, 23).

Obesity and subsequent metabolic disease in adolescent and adulthood have been associated with adverse intrauterine events in the so-called “Developmental Origins of Health and Disease (DOHaD)” (24), even if the underlying mechanism is not well understood (23). The study regarding Dutch Hunger Winter (25) has shown that fetuses, who experienced malnutrition during the first trimester of pregnancy, had a higher risk of cardiovascular complication, including a pro-atherogenic lipid profile and reduced cognitive function. Differently individuals suffering starvation at the end of gestation had a higher risk of glucose intolerance at adult age (15). Heijmans et al. (26) recently have shown a possible link between the Dutch Cohort and epigenetic markers. They have found that individuals who were prenatally exposed to famine during the Dutch Hunger Winter in 1944–45 had, 6 decades later, reduced methylation of the IGF2 imprinted gene, leading to an overexpression of IGF2, compared with their unexposed, same-sex siblings (15, 26).

An overexpression of IGF2 is also caused by a hypermethylation of its imprinting control region IC1. An important study regarding IGF2/H19 IC1 DNA methylation in human models was conduced by Huang et al. (23). They showed that a greater IGF2/H19 IC1 DNA methylation, causing an overexpression of IGF2, was associated with greater subcutaneous fat measures in 17 years old adolescents (23). IGF2/H19 IC1 DNA methylation was not associated with weight, length or head circumference at birth, but was negatively associated with HC between 1 and 10 years (23). Interestingly, patients with Silver Russell syndrome and decreased expression of IGF2 have a striking lack of subcutaneous fat as a part of their clinical phenotype (23).

Other studies underlined the relationship between IGF2/H19 IC1 DNA methylation and the development of overweight/obesity. St. Pierre et al. (27) showed that IGF2 overexpression on placental DNA was associated with fetal metabolic programming of late-onset obesity (27). Perkins et al. (28) hypothesized that epigenetic mechanisms may drive obesity in early childhood, showing greater IGF2/H19 IC1 DNA methylation in overweight or obese 1-year-olds compared to normal weight counterparts (28).

The aim of our study was to investigate the relationship between growth patterns, nutritional determinants, and epigenetic mechanisms.

## Materials and Methods

We enrolled 38 newborns admitted to NICU at University Hospital of Pisa (Italy) from January 2016 to July 2016. Inclusion criteria were: gestational age (GA) at birth <34 weeks and post-menstrual age (PMA) between 36 and 42 weeks at discharge. We excluded infants with congenital malformations or clinical syndromes.

We recorded several variables categorized as prenatal, neonatal, auxological, nutritional, and epigenetic factors (Table 1).

**Table 1:** Variables analyzed.

Prenatal factors	Pregnancy (single, multiple, first or subsequent) Presence of pregnancy complications such as placenta abruption, PROM, pre-eclampsia/eclampsia, gestational diabetes. Delivery (spontaneous, cesarean section) IUGR Antenatal steroids
Neonatal factors	Gender Gestational age SGA for weight and head circumference Preterm birth complications: anemia, IVH, PDA that requires therapy, sepsis, NEC, respiratory failure (days of mechanical ventilation), BPD, ROP, Hypotension Postnatal steroids
Auxological factors	Auxological parameters (weight, length, and head circumference) at birth and at discharge. Weight at 28 days of life (z-scores) Weight at 36 weeks PMA (z-scores) Weight loss (%) Time need to re-gain birth weight EUGR for weight (EUGRw) and for head circumference (EUGRhc)
Nutritional factors	Days of parenteral nutrition Time to reach full enteral feeding Kind of milk (human milk, mixed, formula) Macronutrients concentration in parenteral and enteral feeding during the first week of life
Epigenetic factors	Methylation levels on IC1 at birth and at discharge

Among prenatal factors, we considered z-score of weight and head circumference at birth, during the hospital stay and at discharge, using 2013 Fenton growth chart for preterm infants (29). The use of exact z-scores and SD permit optimal assessment of infants' growth. For each infant in the study population, weight and head circumference z-scores were calculated using the LMS tables (Lambda for the skew, Mu for the median, and Sigma for the generalized coefficient of variation), published by Fenton and Sauve (30). The z-score (Standard Deviation Score) = (X–U)/SD, where X is the individual value, U denotes mean value and SD denotes standard deviation (31). SGA (small for gestational age) was defined as an anthropometric value < −1.28 SD (<10th percentile), referring auxological data to SD or z-score. EUGR was defined the reduction >1 SD (severe EUGR>2 SD) in anthropometric parameters z-score between birth and other measures taken during the hospital stay (7, 8).

Parenteral and enteral feeding was evaluated daily considering the amount of fluids (ml/kg/day), macronutrients (g/kg/day), macroelements (mg or mEq/kg/day) assumed by the newborns. Other infusions (e.g., saline or glucose 5%) received were considered in the daily fluid intake.

The epigenetic analysis was performed on peripheral blood samples in EDTA, collected in two different timepoints (at birth and at discharge). All our evaluations are part of the normal care given to preterm infants and for the evaluation of IC1 methylation we used a small amount of samples, remained after the determination of blood count and/or the cross-tests. DNA extraction was performed using QIAsymphony (Qiagen, Hilden, Germany). Methylation level was evaluated through methylation-specific multiplex ligation-dependent probe amplification (MS-MLPA) on one imprinting control region (ICR) on 11p15. MS-MLPA was performed on genomic DNA with SALSA MS-MLPA kit ME030-c3 (MRC-Holland, Amsterdam, The Netherlands).

Statistical analysis was performed using STATA 12 (Stata Corporation, TX, USA). The distribution of data was tested with the Shapiro-Wilk's test. Comparisons between groups were made by Student's *t*-test for continuous variables and with Odds Ratio (OR) for qualitative variables. Data with skewed distributions were analyzed with a parametric test (Mann-Whitney test). The correlation between two continuous variables was evaluated by linear regression and scatter plots. Values of *p* < 0.05 were considered statistically significant. Informed consent was collected from neonates' parents for data analysis and blood exams.

## Results

**Figure 1:**
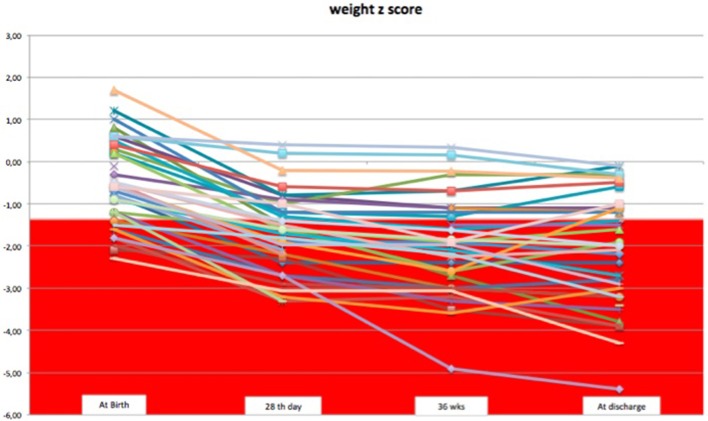
The trend of weight z-score during the hospital stay.

**Figure 2:**
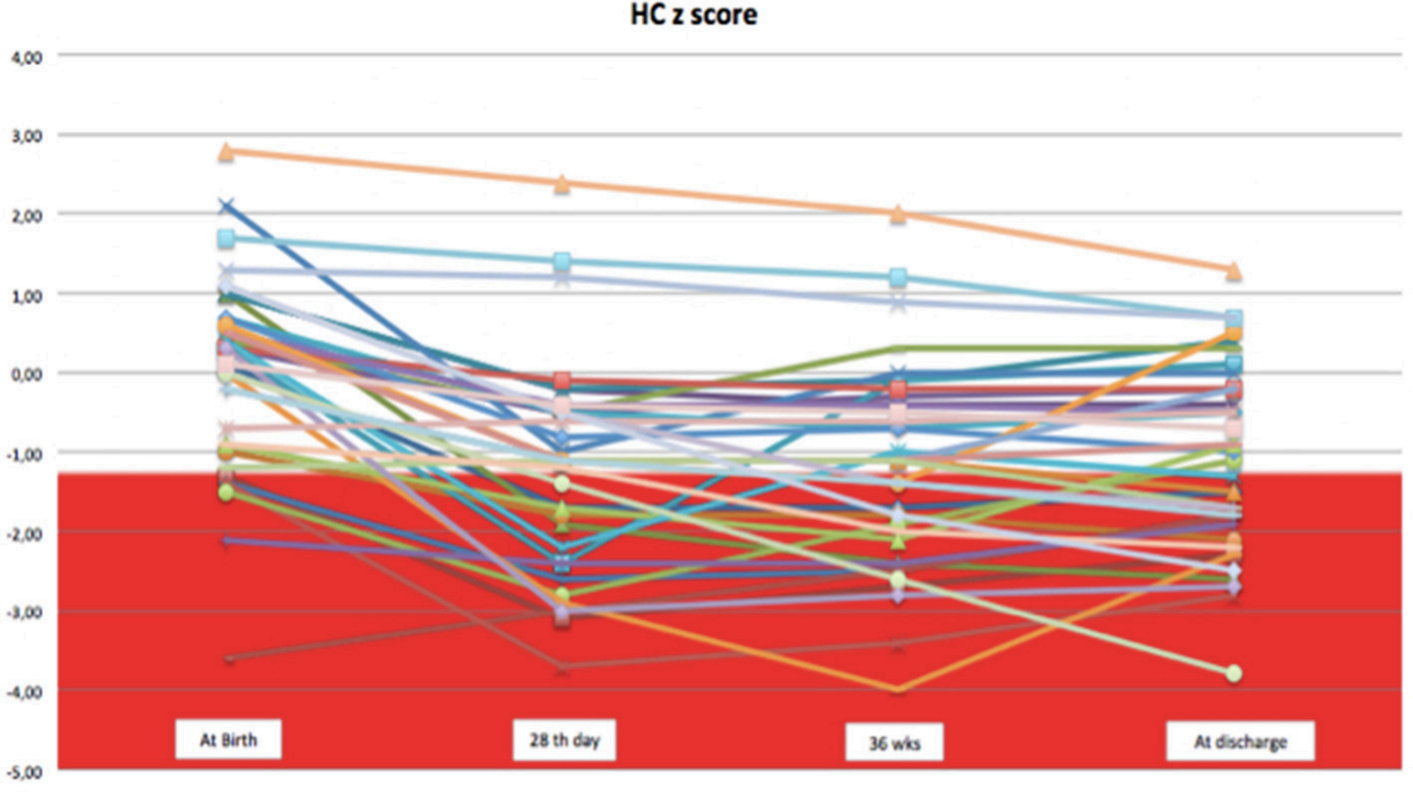
The trend of head circumference z-score during the hospital stay.

We observed a decrease in SD of weight and head circumference mainly during the first 28 days of life. From 1 month of life to discharge, z-score remained almost unchanged, except for few cases (Figures 1, 2).

**Figure 3:**
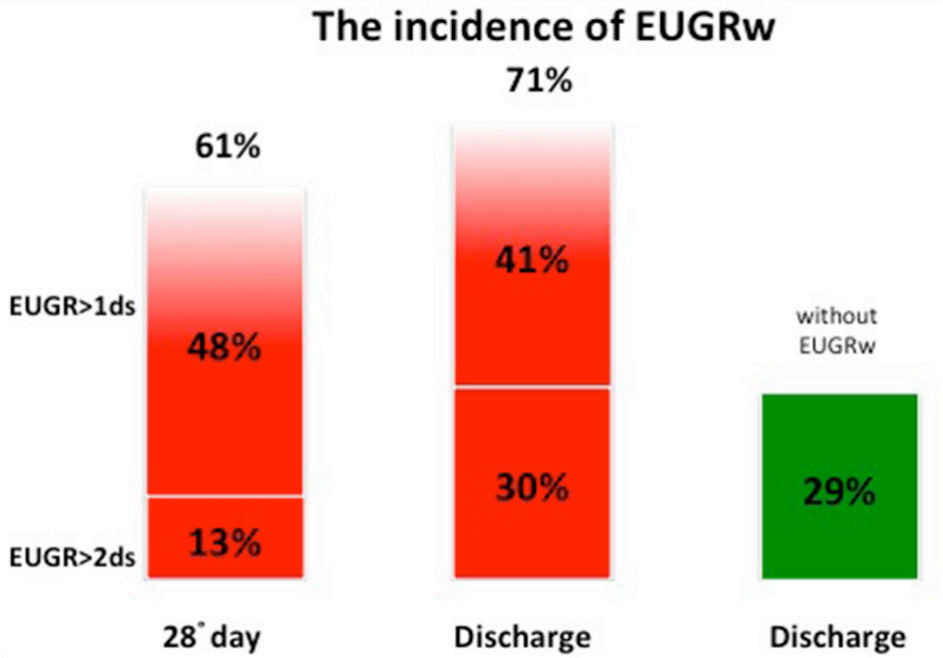
The global incidence of EUGRw during the hospital stay.

**Figure 4:**
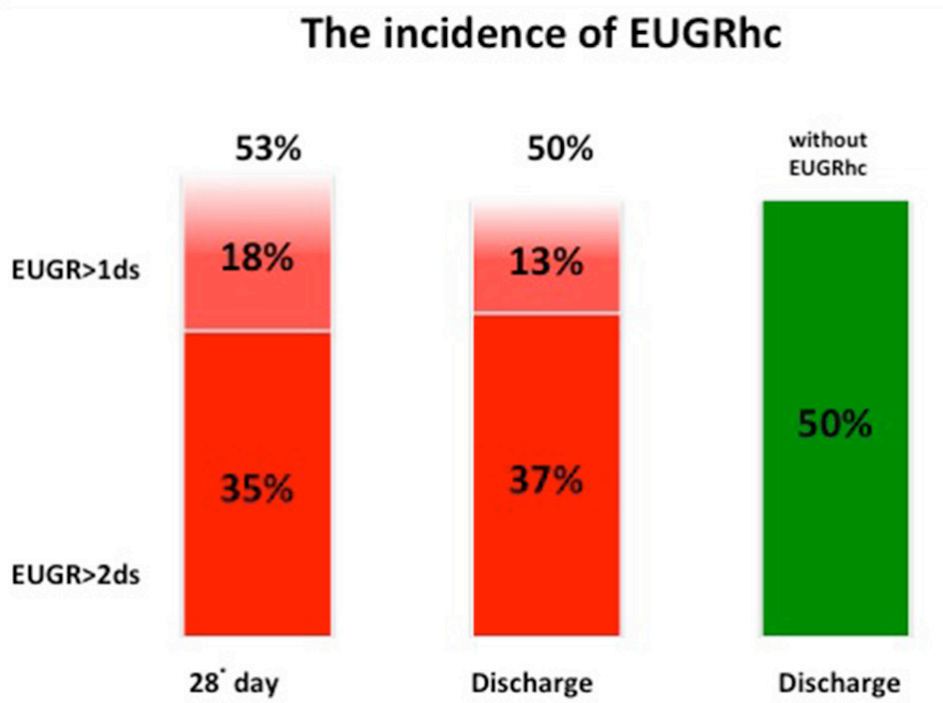
The global incidence of EUGRhc during the hospital stay.

At discharge the incidence of EUGR for weight (w) was 71% and the incidence of EUGR for head circumference (hc) was 50%. Among these EUGR infants, we observed that 30% showed EUGR >2 SD for weight and 37% EUGR >2 SD for head circumference (Figures 3, 4).

Among the prenatal and neonatal factors considered, we observed that several factors (pre-eclampsia, eclampsia, anemia, PDA, BPD, ROP, antenatal, and postnatal steroids use) were more frequently associated to EUGRw, even if the difference is not significant (Table 2). All our cases with IUGR and/or SGA were EUGRw at discharge (Table 2).

**Table 2:** Patients characteristics and variables distribution, predictors of weight growth restriction at discharge.

**Clinical variables**	**Global Incidence**		**EUGRw**	**notEUGRw**	***p*-value**
**PRENATAL FACTORS** ***n*** **(%)**					
Gender (Male)	16 (42%)	**OR** 1.4	12	4	0.46
Cesarean section	32 (84%)	**OR** 0.44	22	10	0.42
First pregnancy	28 (73.7%)	**OR** 0.52	19	9	0.38
Pre-Eclampsia/Eclampsia	11 (28.9%)	**OR** 2.25	9	2	0.30
Gestational diabetes	7 (18.4%)	**OR** 2.8	6	1	0.33
IUGR	4 (10.5%)		4	0	
Antenatal steroids	32 (84%)	**OR** 3	24	8	0.22
**NEONATAL FACTORS**					
Gestational age (mean in weeks)	29 ± 2	**t-test**	29.4 ± 0.43	28.6 ± 0.60	0.31
SGA for weight–*n* (%)	10(26.3)		10	0	
SGA for hc–*n* (%)	6 (15.8)	**OR** 2.27	5	1	0.43
Anemia–*n* (%)	32 (84)	**OR** 1.27	23	9	0.5
Trasfusions–*n* (%)	22 (57.9%)	**OR** 3.5	18	4	0.08
PDA–*n* (%)	8 (21%)	**OR** 3.5	7	1	0.24
Sepsis–*n* (%)	2 (5.7%)		2	0	
NEC–*n* (%)	0				
VMI (mean days)	2 ± 4				
NIVM (mean days)	19 ± 16.2				
BPD–*n* (%)	4 (10.5%)		4	0	
ROP–*n* (%)	5 (13.2%)		4	1	
Hypotension with dopamine treatment need–*n* (%)	5 (13.2%)		4	1	
Postnatal corticosteroids–*n* (%)	10 (26.3%)	**OR** 1.89	8	2	0.38
**AUXOLOGICAL FACTORS**					
Birth weight, BW (g)	1159 ± 358.4	***t-*****test**	1157 ± 76	1166 ± 80	0.94
Birth length (cm)	37.07 ± 3.51	***t-*****test**	37.2 ± 0.74	36.6 ± 0.80	0.63
Birth hc (cm)	26.95 ± 2.45	***t-*****test**	27.03 ± 0.50	26.72 ± 0.63	0.73
Lower weight (g)	976.7 ± 310.4	***t-*****test**	968.4 ± 64	997.8 ± 80	0.79
Days to lower weight (mean)	4.0 ± 1.62	***t-*****test**	4.40 ± 0.32	4.63 ± 0.47	0.69
Days to regain BW (mean)	15.0 ± 4.4	***t-*****test**	14.7 ± 0.87	14.0 ± 1.3	0.70
Weight at discharge (g)	2407 ± 455.1	***t-*****test**	2291.5 ± 88	2690 ± 93	0.01^*^
Length at discharge (cm)	45.5 ± 2.6	***t-*****test**	45 ± 0.51	46.6 ± 0.67	0.08
Hc at discharge (cm)	32.6 ± 1.7	***t-*****test**	32.3 ± 0.34	33.5 ± 0.37	0.04^*^
EUGRhc n (%)	19 (50%)	**OR** 20	18	1	0.001^*^

* *significant p-value < 0.05*.

### Nutritional Factors and EUGR

Among all nutritional factors considered (Table 3), the analysis of median single nutritional supply in the first week of life showed that a greater protein intake decrease the risk of EUGRw. *T*-test showed that infants with EUGRw have received a lower protein intake than newborns without EUGRw (EUGRw 2.56 ± 0.05 g/kg/day vs. notEUGRw 2.86 ± 0.06 g/kg/day ^*^p = 0.005). Furthermore, a low lipid intake had also an important role in postnatal growth restriction (*t*-test: EUGRw 2.03 ± 0.06 g/kg/day vs. notEUGRw 2.38 ± 0.09 g/kg/day ^*^p = 0.005). The different intake of protein and lipid has been observed retrospectively and it is due to the different severity of the clinical conditions.

**Table 3:** Nutritional factors, predictors of weight and head circumference growth restriction at discharge.

**Clinical variables**	**Global Incidence**		**EUGR**	**notEUGR**	***p*-value**
**NUTRITIONAL FACTORS AND EUGRw**					
Days of parenteral nutrition (mean)	21.0 ± 8,52	***t-*****test**	21.66 ± 1.4	19.3 ± 3.4	0.44
Time to reach full enteral feedings (mean)	24 ± 9.8	***t-*****test**	25 ± 1.5	23 ± 3.9	0.57
Human milk n (%)	10 (26.3%)	**OR** 1.89	8	2	0.38
Formula milk n (%)	9 (23.7%)	**OR** 0.2	4	5	0.058
Mixed milk n (%)	19 (50%)	**OR** 2.18	15	4	0.23
Mean total calories intake in first week of life (Kcal/kg/day)	67.94 ± 5.83	***t-*****test**	66.3 ± 1.1	71.3 ± 1.3	0.005^*^
Total glucose intake in first week of life (g/kg/day)	9.44 ± 0.74	***t-*****test**	9.37 ± 0.16	9.6 ± 0.13	0.39
Total lipid intake in first week of life (g/kg/day)	2.13 ± 0.36	***t-*****test**	2.03 ± 0.06	2.38 ± 0.09	0.005^*^
Total protein intake in first week of life (g/kg/day)	2.65 ± 0.30	***t-*****test**	2.56 ± 0.05	2.86 ± 0.06	0.005^*^
**NUTRITIONAL FACTORS AND EUGRhc**					
Days of parenteral nutrition (mean)	21.0 ± 8.52	***t-*****test**	21.9 ± 1.8	20.1 ± 2.1	0.51
Time to reach full enteral feedings (mean)	24 ± 9.8	***t-*****test**	25.3± 2	23.5 ± 2.5	0.57
Human milk n (%)	10 (26.3%)	**OR** 3.1	7	3	0.13
Formula milk n (%)	9 (23.7%)	**OR** 0.4	3	6	0.22
Mixed milk n (%)	19 (50%)	**OR** 0.81	9	10	0.5
Mean total calories intake in first week of life (Kcal/kg/day)	67.94 ± 5.83	***t-*****test**	67.4 ± 1.15	68.4 ± 1.5	0.60
Total glucose intake in first week of life (g/kg/day)	9.44 ± 0.74	***t-*****test**	9.5 ± 0.17	9.37 ± 0.17	0.56
Total lipid intake in first week of life (g/kg/day)	2.13 ± 0.36	***t-*****test**	2.08 ± 0.7	2.17 ± 0.9	0.41
Total protein intake in first week of life (g/kg/day)	2.65 ± 0.30	***t-*****test**	2.58 ± 0.06	2.72 ± 0.07	0.14

* *significant p-value < 0.05*.

### ICR DNA Methylation and Nutritional Factors

**Figure 5:**
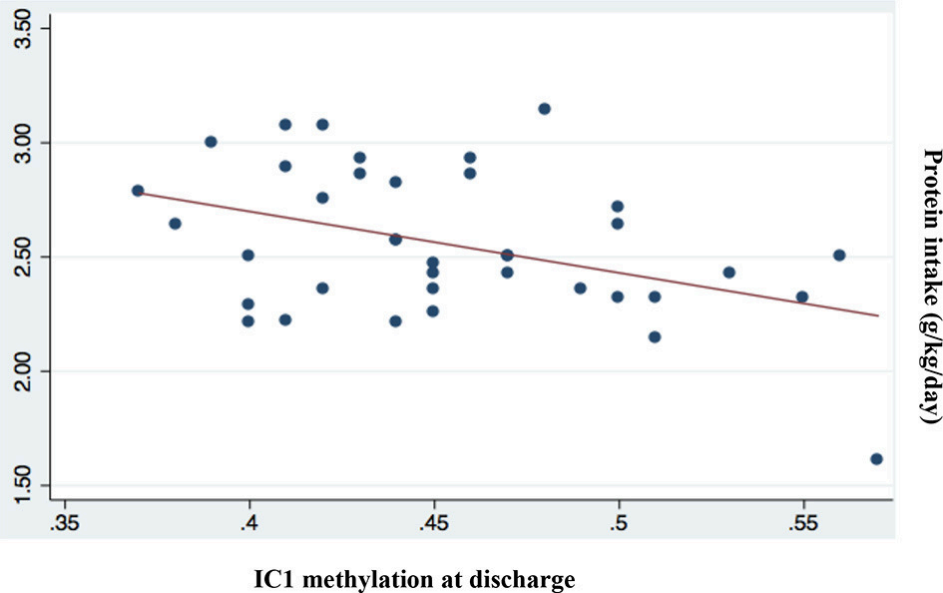
Correlation between IC1 methylation at discharge and protein intake (g/kg/day; *p* = 0.009; *r*^2^ = 0.17).

**Figure 6:**
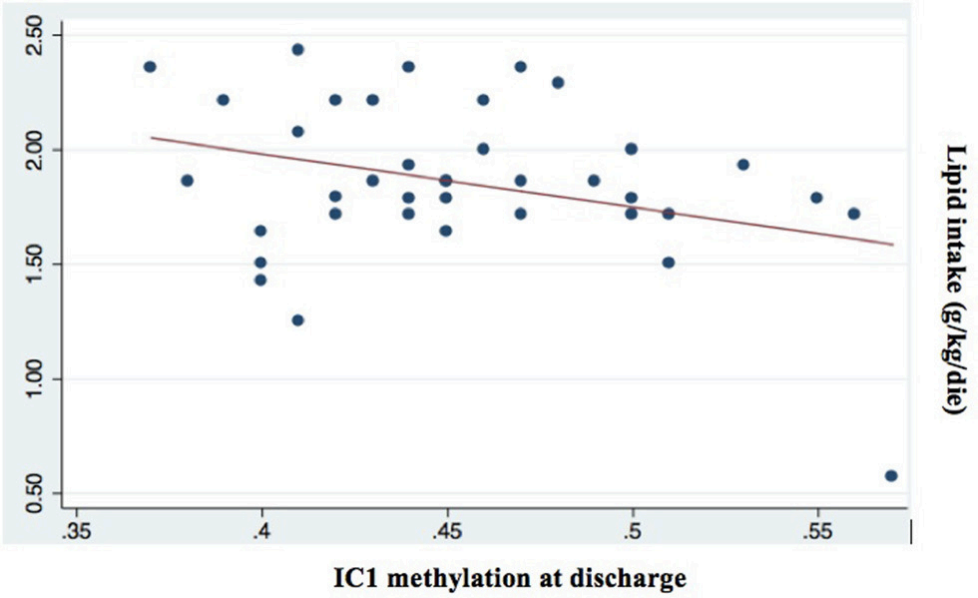
Correlation between IC1 methylation at discharge and lipid intake (g/kg/day; *p* = 0.043; *r*^2^ = 0.11).

Among all nutritional factors considered (Table 4), we observed a negative weak correlation between IC1 methylation levels at discharge and protein and lipid intake in the first week of life (Coef −0.06 [95% CI −0.11 to −0.02] ^*^p = 0.009; *r*^2^ 0.17; Coef −0.05 [95% CI −0.09 to −0.001] ^*^p = 0.043; *r*^2^ 0.11, respectively; Figures 5, 6). These data should be evaluated on a larger case series.

**Table 4:** Nutritional factors and IC1 methylation at discharge.

**Regression IC1 with:**	**Coefficient**	**95% Confidence Interval**	***p*-value**
Liquid total/kg	−0.001	−0.003 to 0.0004	0.13
Total calories	−0.0008	−0.004 to 0.002	0.55
Parenteral calories	−0.002	−0.005 to 0.0006	0.12
Enteral calories	0.001	−0.001 to 0.003	0.37
Calories/Protein	0.01	0.04 to 0.02	0.004^*^
Total glucose	0.013	−0.009 to 0.03	0.22
Parenteral glucose	0.006	−0.02 to 0.03	0.59
Enteral glucose	0.01	−0.13 to 0.03	0.38
Total protein	−0.06	−0.11 to −0.012	0.017^*^
Parenteral protein	−0.06	−0.11 to −0.02	0.009^*^
Enteral protein	0.06	−0.06 to 0.17	0.34
Total lipid	−0.03	−0.07 to 0.02	0.225
Parenteral lipid	−0.05	−0.09 to −0.001	0.043^*^
Enteral lipid	0.01	-0.02 to 0.06	0.37

* *significant p-value < 0.05*.

### ICR DNA Methylation and Auxological Parameters

Regarding the epigenetic analysis we considered first the correlation between IC1 DNA methylation and weight and head circumference at birth and we did not find any significant correlation.

Evaluating the methylation levels between birth and discharge, we observed a dynamic trend. An increased methylation of IC1 at discharge, at least 5% over birth values, was found in 47.3% of our cases. We have considered the IC1 hypermethylation in relation with growth patterns, by Fisher's exact test, and we have found significant correlations. Most of the patients with EUGRw and EUGRhc showed an increased IC1 methylation (>5%) from birth to discharge (*p* = 0.018 and *p* = 0.0028, respectively), respect to newborns without EUGR.

## Discussion

We observed an elevated incidence of EUGR for weight (EUGRw = 71%) and of EUGR for head circumference (EUGRhc = 50%).

Considering weight and head circumference of the infants weekly (29), it was possible to observe that the greatest decrease in the z-score occurred in the first 28 days of life and the greater part of the EUGR newborns at discharge already showed an extrauterine growth restriction in the first month of life (Figures 3, 4). Another important aspect to be assessed is that in the subsequent period, between 28 days and 36 weeks, weight z-score values did not decrease further, except in rare cases. Furthermore, starting from 36 weeks a catch-up growth was more evident in many cases.

An appropriate nutrition is known to be fundamental to promote growth and could be crucial for improvement of postnatal growth (32–37). In our study we confirmed that a low protein and lipid intake had a remarkable impact in postnatal growth restriction (38).

The effect of nutrition on neonatal growth could be associated with epigenetic mechanism as we observed in our data regarding the correlation between methylation and the protein and lipid intake. Some authors underlined the relationship between reduced protein intake and DNA methylation (39–41). Gong et al. (41) found that IC1 DNA methylation increased significantly following maternal low protein diet in a cohort of Sprague-Dawley rats (41). Heijmans and colleagues have shown a possible link between the Dutch Cohort and epigenetic markers (26). They found that the adult people, that suffering starvation during intrauterine life, had an increased expression of IGF2 when compared to matched controls and had a higher risk of cardiovascular complication, including a pro-atherogenic lipid profile, a reduced cognitive function, and also a higher risk of glucose intolerance at adult age (26).

We found only a negative weak association between protein and lipid intake and IC1 methylation and these data should be evaluated on a larger case series (38).

Regarding the results on epigenetic analysis and auxological parameters, Huang's study showed that a greater IGF2/H19 IC1 DNA methylation was associated with greater subcutaneous fat measures (23). Secondly, as IGF2/H19 IC1 DNA methylation was not associated with birth weight, length or HC, but was negatively associated with HC between 1 and 10 years and they hypothesized that the HC may be a more sensitive marker of early life programming of the IGF axis and of fetal physiology than birth size (23).

We considered first the correlation between IC1 methylation and weight and head circumference at birth and we did not find any significant correlation (38). We observed a correlation between reduced postnatal growth of weight and head circumference and the increased levels of IC1 methylation from birth to discharge, according to Huang's study (23, 38).

Postnatal head growth restriction is a relatively common condition in preterm neonates and head circumference correlates with brain volume. Different reports have described an association between reduced head size and neurodevelopmental outcome (42, 43). It was also interesting to note some evidences about the association between hypermethylation of IC1 and head growth restriction in human models (23, 44–46). Furthermore, Pidsley's study showed that epigenetic variation across the IGF2/H19 IC1 has a significant negative relationship with cerebellar mass in mice (47).

## Conclusion

We could consider IC1 hypermethylation, associated to reduce nutritional intake in several human and animal models, as a reprogramming mechanism to promote a catch-up growth, by means of an increase of IGF2 expression. In fact in our study infants with EUGRw and/or EUGRhc had an increase of IC1 methylation from birth to discharge. According to this hypothesis, IC1 methylation could be used as a marker of reduced growth and may guides us to prevent severe EUGR. A persistent IC1 hypermethylation since early life could be responsible for an altered metabolic setting, leading to metabolic and cardiovascular complications in the adult life (26). Our study is a pilot study, but this initial finding should be considered for further validation in a larger case series.

## Ethics Statement

The protocol was approved by the Ethical committee of the University Hospital of Pisa. All subjects gave written informed consent in accordance with the Declaration of Helsinki.

## Author Contributions

MT designed the study, carried out the initial analyses, drafted the initial manuscript, revised it, and approved the final manuscript as submitted. FM carried out the initial analyses, drafted the initial manuscript, and approved the final manuscript as submitted. AM and CC performed genetic analyses, reviewed, and approved the final manuscript as submitted. FL and MC critically reviewed statistical analyses, drafted the initial manuscript, revised it, and approved the final manuscript as submitted. PG conceptualized and designed the study, drafted the initial manuscript, coordinated and supervised data collection, and approved the final manuscript as submitted. All authors approved the final manuscript as submitted and agree to be accountable for all aspects of the work.

### Conflict of Interest Statement

The authors declare that the research was conducted in the absence of any commercial or financial relationships that could be construed as a potential conflict of interest.

## Abbreviations

EUGR: ExtraUterine Growth Restriction
EUGRw: ExtraUterine Growth Restriction for weight
EUGRhc: ExtraUterine Growth Restriction for head circumference
SGA: Small for Gestational Age
IGF2: Insulin-like Growth Factor 2
PMA: PostMenstrual Age
IC1: Imprinting Centre 1
DOHaD: Developmental Origins of Health and Disease
PROM: Prolonged rupture of membranes
IUGR: Intrauterine growth restriction
IVH: Intraventricular hemorrhage
PDA: Patent ductus arteriosus
NEC: Necrotizing enterocolitis
BPD: Broncopolmunary dysplasia
ROP: Retinopathy of premature.
